# Reported Availability, Additional Cost and Use of Methods to Limit Cross‐Contact of Gluten‐Free Foods in Restaurants in a Canadian Large Urban Centre

**DOI:** 10.1111/jhn.70239

**Published:** 2026-03-31

**Authors:** Danielle Defries, Caitlynn Danchuk, Jo‐Ann Stebbing, Jay C. Petkau, Heather Blewett

**Affiliations:** ^1^ Department of Kinesiology and Applied Health University of Winnipeg Winnipeg Manitoba Canada; ^2^ Department of Food and Human Nutritional Sciences University of Manitoba Winnipeg Manitoba Canada; ^3^ Agriculture and Agri‐Food Canada, Government of Canada, Route 100 Morden Manitoba Canada; ^4^ Canadian Centre for Agri‐Food Research in Health and Medicine Winnipeg Manitoba Canada

**Keywords:** availability, cost, cross‐contact, gluten‐free, restaurants

## Abstract

**Background:**

The availability and cost of gluten‐free (GF) products in grocery stores and online have increased in recent years, but less is known about availability and cost of GF menu items in restaurants, as well as whether methods are being implemented to limit cross‐contact (CC) of GF with gluten‐containing menu items. The purpose of this study was to assess the reported availability, additional cost and use of methods to limit cross‐contact (CC) of GF items in restaurants in Winnipeg, Canada.

**Methods:**

Restaurant staff were asked over the telephone if they served GF items, if there was an additional cost for these items and if they used methods to limit gluten CC. Chi‐squared (*X* ^2^) tests were used to test for differences in the proportion of restaurants reporting serving GF items, charging extra and using methods to limit CC based on service type, locality, and ethnic cuisine type. A p‐value of < 0.05 was considered significant.

**Results:**

Of the 548 restaurants surveyed, 69% reported offering GF items. A higher proportion of full‐service restaurants (73%) reported offering GF foods compared to quick‐service restaurants (55%). A lower proportion of full‐service restaurants that served GF foods reported a surcharge (22%) compared to quick‐service restaurants (48%). Similarly, a lower proportion of full‐service restaurants that served GF foods reported using methods to limit CC (79%) compared to quick‐service restaurants that served GF foods (90%). A higher proportion of chain restaurants reported GF menu options (78%), additional charge for GF foods (41%) and using methods to limit CC (89%) compared to local restaurants (67%, 22%, 78%, respectively). The type of cuisine significantly affected the proportion of restaurants reporting GF item availability, surcharges and whether methods were used to limit CC.

**Conclusions:**

Availability of GF foods, cost and use of procedures to limit gluten CC vary based on type of service, locality, and cuisine served.

## Summary

A survey of 548 restaurants revealed that the availability and pricing of gluten‐free menu items, as well as procedures to limit cross‑contact varied widely depending on service type (full‑service vs. quick‑service), locality (local vs. chain), and cuisine (Asian, Chinese, East Indian, European, Japanese, Latin American, Middle Eastern, Mediterranean, American/Canadian).

## 1 Introduction

Celiac disease (CD), gluten ataxia, dermatitis herpetiformis, wheat allergy, and non‐celiac gluten sensitivity are gluten‐related disorders that require individuals to adopt a gluten‐free (GF) diet to manage symptoms, minimize intestinal damage, and prevent secondary conditions such as nutrient deficiencies [1, 2, 3, 4, 5]. Complete elimination of gluten from the diet, although straightforward in theory, is challenging in practice and imposes a significant collective economic, health, and psychosocial burden on those following a GF diet. For example, GF versions of common staple foods are more expensive and of lower nutritional quality compared to similar gluten‐containing foods [1, 2, 3]. Beyond the financial and nutritional implications, strict adherence to a GF diet requires individuals to make major lifestyle changes that can affect their quality of life [2, 4]. Social settings involving food are an area where those following a GF diet commonly report experiencing difficulties and negative emotions, mainly due to restricted food choices and the need to explain and justify their dietary needs [5, 6].

Dining out in restaurants is a social activity that is drastically altered for those following a GF diet and has also been reported as one of the largest perceived barriers to diet adherence [5, 7]. Lack of GF menu items, low confidence in restaurant staff's knowledge, or fear of accidentally consuming gluten due to cross‐contact are some of the major concerns that often lead to anxiety, feelings of isolation, or stigma experienced by those following a GF diet while dining in a restaurant [5, 8, 9]. It is not surprising, then, that patients with CD report dining out less [10, 11], and a high percentage report avoiding dining out completely [5]. Knowledge of CD and other gluten‐related disorders appears to be increasing within the restaurant industry [12]; however, this knowledge can be highly variable in restaurant staff and dependent on the level of training chefs and cooks have received [9, 13].

The availability of GF products in grocery stores and online has increased in recent years [14, 15], but less is known about availability of GF menu items in restaurants, as well as the methods used by the restaurant industry to ensure safety of GF menu items. There is no Canadian legislation on allergen labeling in restaurants, therefore consumers must rely on the information provided by restaurant staff. The main objective of the current study was to assess the reported availability of GF items in restaurants in Winnipeg, Manitoba, a major city within the prairie provinces of Canada. We also sought to determine if restaurants that reported offering GF food items charged more for these items and if they implemented any procedures to limit cross‐contact of GF foods with gluten‐containing foods.

## 2 Materials & Methods

### 2.1 Population Selection

Restaurants (*N* = 676) were identified by searching the online Yellow Pages and Zomato for Winnipeg area restaurants and filtered by cuisine in January 2021. For restaurants with multiple listings, only the first location was recorded. All restaurants were contacted. In total, information was collected from 548 unique restaurants. The complete list of restaurants, along with their address, phone number, website, and restaurant classification, is provided in the Supplementary Table “Listing of Winnipeg restaurants with classifications.”

Restaurants were categorized based on locality, service type, and ethnic cuisine type. Restaurants were considered local if their locations were exclusively within Winnipeg, whereas restaurants with multiple locations across or outside of Canada were considered chain restaurants. Full‐service restaurants (FSR) were those that employed serving staff to take orders and deliver food to patrons seated at a table, whereas quick‐service restaurants (QSR) were those where patrons ordered and received their food at a counter. Ethnic cuisine type was based on global region of the origin of the food, with categories defined as American/Canadian, Italian/Greek/Mediterranean, East Indian, Chinese, Japanese, European (including French, Danish, British, and Irish cuisine), Asian (including Vietnamese, Thai, Korean, Filipino, and Mongolian cuisine), Middle Eastern (including Middle Eastern, Lebanese, Georgian), Latin American (including Caribbean, Mexican, Brazilian, Argentinian, and Salvadoran cuisine), and African. Restaurant classification was performed independently by three members of the research team; in situations where restaurants were classified differently amongst members, consensus was reached through discussion.

### 2.2 Data Collection

Each restaurant identified from the above search was contacted by telephone.

If possible, study staff spoke to the restaurant manager, but if the manager was not available they would speak to the staff member who answered the phone. A series of 3 questions were asked: “Are GF menu items offered at your restaurant (yes or no)?”, “If GF items are offered, is there a surcharge for GF items (yes or no)?”; “If you serve GF items in your restaurant, do you have procedures in place to prevent cross‐contact from gluten containing menu items (yes or no)?”. If restaurants reported that there were procedures in place to limit cross‐contact, the procedures described by staff were categorized into 1 of 3 groups: 1=Wash hands/change gloves/aprons and any food preparation surfaces/containers/utensils before or in between preparing regular food; 2=Separate utensils/cooking dishes (i.e., Frying pan, griddle, pot, colander, dedicated fryer or fresh aluminum foil); 3=Food prepared in an area separate from the regular flow of the kitchen.

### 2.3 Statistical Analysis

Chi‐squared (*X* ^2^) tests were used to examine the significance of differences in the proportion of restaurants reporting availability of GF menu items, the presence of a surcharge for GF items, the existence of protocols to reduce cross‐contact, and the type of protocols used. Sub‐analysis was conducted to examine if these variables differed based on restaurant type, locality, and ethnic cuisine type. Results with a *p*‐value of < 0.05 were considered statistically significant. Comparisons amongst the ethnic cuisine types were made using multiple *X* ^2^ tests with the Bonferroni method applied to account for multiple comparisons; the adjusted threshold for significance was *p* < 0.001. The likelihood of GF options being more available and the likelihood of GF menu items incurring a higher cost based on restaurant service type or locality of restaurant was tested using odds ratios. Statistical analyses were performed with SAS version 9.4 (SAS Institute Inc., Cary, NC, USA) and Prism.

## 3 Results

**Figure 1:**
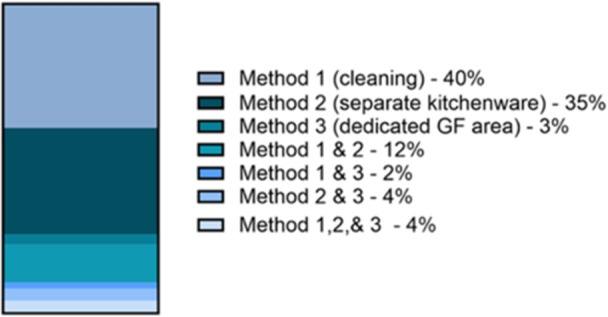
Percentage of all restaurants reporting different methods used to prevent gluten cross‐contamination. Cleaning refers to washing cookware, utensils, hands, or changing gloves or aprons in between preparing gluten‐containing and GF foods. Separate kitchenware refers to the use of separate pots, pans, baking sheets, appliances, fryers, utensils, etc. for preparing GF items. Dedicated GF area refers to the existence of a separate area of the kitchen where only GF foods are prepared. Where more than one method was reported, categories representing the combination of methods used were created.

Of the 548 unique restaurants surveyed, 69% reported offering GF menu items. A surcharge for GF items was only reported by 26% of restaurants with GF menus items. 80% of restaurants that reported offering GF items reported using methods to limit contact of GF items with gluten. In our study, we classified methods used to prevent gluten CC into three different categories: (1) cleaning; (2) separate kitchenware; (3) dedicated GF area. When asked to describe the nature of the methods used to prevent gluten CC, most restaurants that served GF foods (78%) reported using only one method to prevent gluten CC. Use of two of the above‐mentioned methods was reported by 18%, and 4% of all GF food serving restaurants surveyed reported using all 3 of the methods (Figure 1). Methods involving cleaning, such as washing of hands and/or utensils was the most prevalent method (58%), followed by use of separate kitchenware to prepare GF items (55%), and existence of a separate area of the kitchen dedicated only to preparing GF items (13%).

FSRs constituted the majority of restaurants surveyed, with 435 (79%) restaurants classified as FSRs and 113 (21%) classified as QSRs (Table 1). There was a significant relationship between restaurant service type and reported availability of GF menu options: 73% of FSRs reported offering gluten‐free items, while only 55% of QSRs reported the same, *X* ^2^(1, *N* = 548) = 13.2, *p* < 0.001. The odds of FSR reporting offering a GF menu item were two times the odds of QSRs reporting the same (OR = 2.18, 95% CI 1.15–3.34). Likewise, there was a significant relationship between service type and reported higher cost of GF items, with 22% of FSRs and 48% of QSRs reporting a surcharge for their GF items, *X* ^2^(1, *N* = 375) = 19.1, *p* < 0.0001. The odds of QSRs reporting imposing a surcharge for GF items were slightly more than 3 times the odds of FSRs reporting the same (OR = 3.38, 95% CI 1.95–5.83). The percentage of restaurants reporting employing procedures to limit cross‐contact with gluten was higher in QSR (90%) versus FSRs (79%), a relationship which was significant, *X* ^2^(6, *N* = 302) = 15.8, *p* = 0.02. The odds of QSRs reporting the use of procedures designed to limit gluten CC were 2.5 times of FSRs reporting the same (OR = 2.53, 95% CI 1.08–5.75).

**Table 1:** Prevalence of availability, additional cost, and methods to limit cross‐contact (CC) of gluten‐free foods with gluten in quick service restaurants compared to full‐service restaurants.

	Availability # Yes/No (% Yes)	Additional cost # Yes/No (% Yes)	Methods to limit CC # Yes/No (% Yes)
Quick service	62/51 (55%)	30/32 (48%)	56/6 (90%)
Full service	316/119 (73%)	68/245 (22%)	246/67 (79%)
*X* ^2^ (*p*‐value)	13.2 (< 0.001)	19.2 (< 0.001)	4.54 (*p* = 0.03)

When examining restaurants based on their locality, only 98 (18%) were considered chain restaurants while 450 (82%) were considered local (Table 2). There was a significant relationship between restaurant locality and availability of GF items, with 78% of chain restaurants and 67% of local restaurants reporting that they offered GF foods, *X* ^2^(1, *N* = 548) = 4.10, *p* = 0.04. The odds of chain restaurants reporting offering GF items were 1.7 times the odds of local restaurants reporting the same (OR = 1.69, 95% CI 1.03–2.79). There was also a significant relationship between restaurant locality and additional cost for GF items, with 41% of chain restaurants and 22% of local restaurants that offer GF items reporting a higher cost for these items, *X* ^2^(1, *N* = 375) = 10.6, *p* = 0.001). The odds of chain restaurants reporting an additional cost for GF items were almost 2.4 times the odds of local restaurants reporting the same (OR = 2.39, 95% CI 1.40–4.03). Use of procedures to limit gluten CC was reported by 89% of chain restaurants but only 78% of local restaurants, X2(1, *N* = 375) = 4.86, *p* = 0.03. The odds of chain restaurants reporting using procedures to limit gluten CC were 2.4 times the odds of local restaurants reporting the same (OR = 2.36, 95% CI 1.10–4.98). There was no association between restaurant locality and the type of methods used to limit CC.

**Table 2:** Prevalence of availability, additional cost, and methods to limit cross‐contact (CC) of gluten‐free foods with gluten in local compared to chain restaurants.

	Availability # Yes/No (% Yes)	Additional cost # Yes/No (% Yes)	Methods to limit CC # Yes/No (% Yes)
Local	302/148 (67%)	67/232 (22%)	234/65 (78%)
Chain	76/22 (78%)	31/45 (41%)	68/8 (89%)
*X* ^2^ (*p*‐value)	4.10 (0.042)	10.6 (0.0011)	4.86 (0.028)

Table 3 presents data on the proportion of restaurants reporting offering GF menu items based on ethnic origin of cuisine. There was a significant relationship between ethnicity of cuisine and reported availability of GF items, *X* ^2^(1, *N* = 548) = 36.3, *p* < 0.0001). Reported availability of GF menu items was lowest in restaurants offering African cuisine (50% of African restaurants surveyed), and highest in restaurants serving Latin American cuisine (95% of Latin American restaurants surveyed). However, statistically significant differences were only observed between Chinese and East Indian restaurants, and American/Canadian and East Indian restaurants (Table 3). There was also a significant association between ethnicity of cuisine and reported higher cost for GF items, *X* ^2^(1, *N* = 375) = 68.8, *p* < 0.0001). The proportion of European (46%), Mediterranean (42%), and American/Canadian (46%) restaurants charging extra for GF items was significantly higher than Asian (5%), Chinese (10%), and East Indian (0%) restaurants (Table 3). Additionally, the proportion of American/Canadian restaurants charging extra for GF items was significantly higher than Japanese restaurants charging extra for GF items (13%). When examining use of methods to limit gluten CC, there was a significant relationship between ethnicity of cuisine and reported use of methods to limit gluten CC, *X* ^2^(1, *N* = 375) = 23.5, *p* < 0.005), with specific statistical differences observed between the proportion of European restaurants (100%) and American/Canadian restaurants (87%) using methods compared to Chinese restaurants (62%).

**Table 3:** Prevalence of availability, additional cost, and methods to limit cross‐contact (CC) of gluten‐free foods with gluten in restaurants of different ethnic origin of cuisine.

	Availability # Yes/No (% Yes)	Additional cost # Yes/No (% Yes)	Methods to limit CC # Yes/No (% Yes)
African	4/4 (50%)	0/4 (0%)	3/1 (75%)
Asian	45/26 (63%)	2/42 (5%)	36/8 (82%)
Chinese	63/40 (61%)	6/57 (10%)	39/24 (62%)
East Indian	17/6 (74%)*	0/17 (0%)	12/5 (71%)
European	13/1 (93%)	6/7 (46%)^‡,*,§^	13/0 (100%)*
Japanese	47/10 (82%)	6/39 (13%)	38/7 (84%)
Latin American	19/1 (95%)	5/14 (26%)	16/3 (84%)
Middle Eastern	10/1 (91%)	0/10 (0%)	7/3 (70%)
Mediterranean	33/3 (92%)	14/19 (42%)^‡,*,§^	27/6 (82%)
American/Canadian	127/78 (62%)^†^	59/68 (46%)^‡,*,§,ǁ^	111/16 (87%)*
*X* ^*2*^ (*p*‐value)	36.3 (< 0.001)	68.8 (< 0.001)	23.4 (0.005)

*Note:* Availability column: * denotes statistically different from Chinese; † denotes statistically different from Mediterranean (*p* < 0.001).

Cost column: ‡ denotes statistically different from Asian; * denotes statistically different from Chinese; § denotes statistically different from East Indian; ǁ denotes statistically different from Japanese (*p* < 0.001).

CC column: * denotes significantly different from Chinese (*p* < 0.001).

## 4 Discussion

Gluten‐related disorders, including CD, non‐celiac gluten sensitivity, and wheat allergy, require individuals to adopt a gluten‐free lifestyle and eliminate foods containing gluten from their diet. The prevalence of dietary gluten avoidance in Canada and the United States is similar, with recent estimates at 1.9% and 2.1% of adults, respectively [16, 17]. The current study sought to gather information related to reported availability, cost, and preparation of GF foods in restaurants in Winnipeg, a large, multicultural urban centre situated in a region of Canada where the prevalence of gluten avoidance is higher than the national average at 2.4% of adults [16]. Across Canada, a significant number of adults who follow a GF diet state that they have few options for GF foods while dining out, or find their choices limited due to a fear of inadvertently consuming gluten [18]. Studies have also reported a relationship between availability of GF items in restaurants and adherence to a GF diet [19]. Given the important role of dining out in social experiences and the social domain of quality of life, information on the landscape of GF dining in restaurants may help inform dining decision for those adhering to a GF diet. This information may also assist the restaurant industry and policy makers in identifying areas where GF dining may be improved.

While most restaurants surveyed in the current study reported having GF options available for patrons, it was still surprising to find that just over 30% of the restaurants surveyed did not offer GF items. With the rising incidence of CD and gluten‐related disorders, and increased general awareness of gluten‐free diets, expanding menus to include GF options would presumably attract a larger customer base, better serve existing customers, and potentially enhance profits [20, 21, 22]. Several factors may influence a food service establishment's decision to offer GF items, including cost, availability of GF ingredients, customer profile, resources such as space and experienced staff, and knowledge of gluten‐related disorders. In the current study, FSRs were twice as likely to report offering GF items in their establishments than QSRs. This is consistent with findings from the Canadian 2024 State of Celiac Survey, where a large proportion of participants reported experiencing difficulties finding GF options in fast food restaurants compared to other types of food service establishments [18]. Although the reasons were not explored in the current study, lower availability of GF foods in QSRs may be due to the standardized nature of menu items at QSRs, or the need for extra time to prepare GF items that is not consistent with quick service models. In addition, risk of accidental cross‐contact may be a reason fast food chains or QSRs may refrain from providing GF options. With high employee turnover and younger, less experienced employees, maintaining stringent staff education standards consistently over time may be difficult to guarantee, and the risk of accidentally exposing a customer to gluten may be perceived as outweighing the benefits of providing GF options [23]. Additionally, QSRs may not have resources such as additional space or separate utensils to support safe preparation of GF foods. Future studies should explore what restaurant owners and managers perceive as the barriers to offering GF foods in their establishments.

In our sample of restaurants, more chain than local restaurants reported offering GF options. This may initially seem to contradict our results for availability of GF items based on service type, as the term “chain” implies “fast food” or “QSRs,” but these terms are not always synonymous. In our study, restaurants were classified as chain if they had more than one location across Canada, which could include multi‐location FSRs. Regardless, the fact that more chain restaurants reported offering GF items may reflect a larger purchasing power to source affordable GF items from bulk suppliers, resulting in a lower cost of offering GF foods. It is well‐established that GF versions of staple foods and ingredients, including flour, pasta, breads, and cereals are more expensive than comparable gluten‐containing foods [1, 2]. Local restaurants, who may not have access to bulk purchasing, may be less inclined to offer GF items due to a higher cost. Interestingly, however, the percentage of local restaurants that reported imposing a surcharge for GF items was almost half that of chain restaurants, suggesting that local restaurants are absorbing the higher costs associated with offering GF items. Overall, only 26% of restaurants offering GF foods reported imposing a surcharge for GF items, reflecting the higher cost for many GF staple foods [1, 24, 25]. However, these results should be interpreted with caution, as some restaurants may distribute the higher food costs of GF foods across all menu items versus imposing a charge for an individual GF item.

The current study also uncovered relationships between ethnic origin of food served and availability GF foods. African restaurants had the lowest positive response rate for availability of GF foods; however, the small sample of African restaurants may have skewed this result and made it difficult to extrapolate these findings to the wider category of African restaurants. Further supporting this is the fact that African cuisine has a relatively large number of staple foods that are naturally GF, including maize, cassava, rice, millet, yams, plantains, beans, and lentils [26]. Similarly, Chinese and Asian restaurants, whose cuisine includes naturally GF staple foods such as rice, vegetables, seafood, and tofu, also had a relatively low percentage of respondents offering GF items. This may be due to the use of soy sauce, which contains wheat, in several Chinese and Asian dishes, which may introduce gluten to naturally‐GF foods. Restaurants serving American/Canadian cuisine also had a relatively low percentage of restaurants reporting availability of GF foods, which is not surprising given the emphasis on products made from wheat flour, such as bread, pasta, noodles, crackers, and bakery products, that tend to be common in American/Canadian cuisine [27]. Further, sauces or gravies in American/Canadian cuisine may be thickened using wheat flour, which would introduce gluten to these items. Regardless of the nature of staple foods, restaurants serving foods from different areas of the world may choose not to offer GF items because they cannot guarantee that their foods will be GF, especially if gluten is present in sauces or other ingredients.

Having GF options while dining out is an important step towards lessening the social impacts of living GF; however, patients with gluten‐related disorders should also feel confident about the integrity of GF meals they are consuming in restaurants. In the current study, 80% of restaurants that reported offering GF items also reported employing methods to limit gluten CC, such as washing utensils and hands between preparing gluten‐containing and GF foods, using separate utensils for gluten‐containing and GF foods, having a dedicated area for preparing GF foods, or combinations of these methods. Our data showed that, although a lower percentage of QSRs reported offering GF items, the percentage of QSRs with established methods to prevent gluten CC was higher than that of FSRs. This may be explained by the standardized and protocol‐driven nature of operations within QSRs. We also observed differences in the percentage of restaurants reporting the use of methods to limit gluten CC based on restaurant locality and restaurants with different ethnic cuisines, with European and American/Canadian restaurants having the highest positive response rates for use of such methods. Many individuals on a GF diet report a lack of confidence in restaurant staff's knowledge of safe food handling and preparation of GF foods, leading them to avoid dining out altogether [18]. Substantiating these concerns are studies reporting the presence of gluten in a high proportion of foods labeled or marketed as GF [28, 29], which may occur through accidental gluten contact [30]. Although there is currently no Canadian legislation on allergen labeling in restaurants, several guidelines have been created to assist food service establishments in preparing safe GF food that focus on preparation steps where gluten transfer may occur [31, 32]. Most, if not all, of these guidelines recommend practices such as using separate utensils when preparing GF food, washing utensils between preparing GF and gluten‐containing food, using separate water for boiling or oil for frying, preparing GF foods in a dedicated space, and using squeezable condiment bottles. Most guidance documents are tailored to large commercial kitchens and production areas; however, the U.S. National Celiac Association has produced an infographic with guidelines for gluten‐free meal preparation specifically for restaurants [33].

Certain steps within the food preparation process may be more vulnerable to gluten transfer than others. A small number of recent studies have shown minimal gluten transfer (defined as less than 20 ppm gluten) when shared wooden spoons, colanders, knives, or toasters are used to prepare GF and gluten‐containing foods [34, 35]. However, given the small sample sizes, the limited protocols tested compared to the wide number of culinary practices in the real world, and the controlled environments in which the studies were performed, it is premature to conclude that shared utensils pose no risk of gluten CC. Furthermore, using the same cooking water for GF and gluten‐containing pasta, or using the same ladle to serve GF and gluten‐containing pasta immediately after cooking does introduce an appreciable level of gluten into GF foods [35]. Still other studies have shown that gluten transfer can be prevented by cleaning shared utensils between contact with gluten‐containing and GF foods [34, 35]. Additional studies with testing protocols that reflect real‐world restaurant kitchen practices using a variety of utensils made from multiple material types (e.g. plastic, stainless steel) must be conducted to provide robust evidence‐based food preparation practices to minimize gluten transfer.

In our study, cleaning was the most frequently reported method used to prevent gluten CC, with 60% of all restaurants reporting use of this method. While 13% of our responding restaurants indicated they use a separate area of their kitchen to prepare GF foods, further consideration to the degree of separation may be required depending on the type of food being prepared. One pilot study demonstrated that GF and regular food preparation areas must be separated by a minimum of 2 meters when wheat flour is being handled to prevent significant gluten contact [36]. This is not surprising, as working with flour can create airborne flour dust that may transfer to surfaces or utensils in adjacent areas. We did not ask our participants for details on the distances between food preparation areas in their kitchen, but given the wide range of restaurant kitchen sizes, it is possible that restaurants may not have the space to meet these requirements. Even if restaurant kitchens have a separate area for preparing GF foods, cleaning protocols and other methods to prevent gluten transfer should still be in place as a precautionary step. Overall, despite a handful of preliminary studies that show low risk of gluten transfer from sharing utensils and appliances, it is still necessary to employ all possible precautionary practices to prevent gluten transfer in restaurant kitchens [37].

While the current study provides insight into the state of dining out while following a GF diet, there are several limitations that must be considered when interpreting the results. First, results are based on self‐reported data from restaurant staff, and although we aimed to speak with management or owners, that was not always possible. Overall, there appears to be a gap in training and practices for safe food handling for allergens and gluten‐related disorders, and while we did not ask if the staff we were speaking to had training, we are not aware of the knowledge of GF practices by the staff member to which we spoke [38]. Future studies comparing restaurant staff knowledge to actual availability and safe food handling practices for GF foods would be an interesting addition to the data. We did not distinguish between foods that are naturally GF and options to substitute common foods that contain gluten with a GF alternative (e.g. substituting a lettuce wrap in place of a hamburger bun). Again, depending on the knowledge of the staff we spoke with, they may be aware only of GF alternatives to gluten‐containing foods versus foods that are naturally gluten free. As mentioned previously, wording our question on additional cost of GF item to consumer as “surcharge” may have only captured additional costs for specific GF item substitutions; however, could there be situations where the additional cost of GF items is incorporated into the higher cost for all menu items. Data were collected during the COVID‐19 pandemic at a time when restaurants may have changed normal operations (e.g. streamlining menus or switching to take out options only) that could have affected GF offerings, and therefore our results may be different than those obtained post‐pandemic. Finally, our results were limited to one urban centre; patterns of GF availability, cost, and methods to ensure safety while dining out may be different in smaller cities or rural areas.

## 5 Conclusion

In conclusion, this study provides insight into the landscape of GF dining in an urban centre, setting a baseline so that policy makers and those in the restaurant industry can improve the current situation. Potential changes may include staff training (i.e. standard operating procedures surrounding the preparation of GF foods), menu development (i.e. create fewer opportunities for cross‐contact), transparent menus (i.e. separate GF menus or using symbols), or participation in a certification program. Given the role of dining out in social and emotional dimensions of quality of life, this information may assist GF diners in making restaurant choices that enhance their restaurant dining experience and increase participation in social gatherings outside of the home.

## Author Contributions

D.D. statistically analyzed the data and wrote the first draft of the article. C.D. and J.S. collected the data and reviewed the manuscript. J.P. assisted in data analysis and reviewed the manuscript. H.B. formulated the research question, designed the study, advised on the statistical analysis of the data and edited the manuscript.

## Funding

The authors have nothing to report.

## Ethics Statement

The authors confirm that the data in this manuscript is our own original work that has not been previously published. All authors have reviewed the contents of this manuscript and agreed to its submission to J Hum Nutr Diet. This work was exempt from Research Ethics Committee review. The author's institutional Research Ethics Review Committees follow the Tri‐Council Policy Statement: Ethical Conduct for Research involving Humans (TCPS 2), and article 2.1 explains that review is not required when interaction with individuals during the ordinary course of their employment, who are not themselves the focus of the research, occurs in order to collect information about their organization.

## Conflicts of Interest

HB has been diagnosed with celiac disease and follows a gluten‐free diet.

## Supporting information

JHND_supplemental material_Winnipeg Restaurants.
